# A preliminary study on plasma markers across cognitive stages and links to a history of mild traumatic brain injury

**DOI:** 10.1177/13872877251325757

**Published:** 2025-03-21

**Authors:** Christian LoBue, Barbara E Stopschinski, Nil Saez Calveras, Amber Salter, Doug Galasko, Chris Giza, C Munro Cullum, Peter M Douglas, John Hart

**Affiliations:** 1 Department of Psychiatry, University of Texas Southwestern Medical Center, Dallas, TX, USA; 2 Department of Neurological Surgery, University of Texas Southwestern Medical Center, Dallas, TX, USA; 3 Peter O’Donnell Jr. Brain Institute, University of Texas Southwestern Medical Center, Dallas, TX, USA; 4 Department of Neurology, University of Texas Southwestern Medical Center, Dallas, TX, USA; 5 Center for Alzheimer's and Neurodegenerative Diseases, University of Texas Southwestern Medical Center, Dallas, TX, USA; 6 Department of Neurosciences, University of California, San Diego, La Jolla, CA, USA; 7 Department of Pediatrics, University of California, Los Angeles, CA, USA; 8 Department of Neurosurgery, UCLA Steve Tisch BrainSPORT Program, University of California, Los Angeles, CA, USA; 9 Department of Molecular Biology, University of Texas Southwestern Medical Center, Dallas, TX, USA; 10 Hamon Center for Regenerative Science and Medicine, University of Texas Southwestern Medical Center, Dallas, TX, USA; 11 School of Behavioral and Brain Sciences, University of Texas at Dallas, Dallas, TX, USA

**Keywords:** Alzheimer's disease, brain injury, blood biomarker, cognitive dysfunction, concussion, dementia of the Alzheimer's type, tau

## Abstract

Potential implications of a history of mild traumatic brain injury (mTBI) during aging are understudied. Seven plasma markers were measured in matched participants having normal cognition, mild cognitive impairment (MCI) and dementia of the Alzheimer's type (DAT) with and without a history of mTBI. Phosphorylated tau_181_ showed a moderate effect size for being greater in mTBI + individuals having MCI and DAT, and effect sizes for lower amyloid-β 42/40 and higher neurofilament light were seen for mTBI + DAT individuals. This preliminary report shows a potential role of plasma-derived markers in detecting associations between mTBI history and the development of Alzheimer's disease and related disorders.

## Introduction

Mild traumatic brain injury (mTBI) often occurs from rapid head acceleration/deceleration, and preclinical experiments show that axonal connections and the blood-brain barrier can be disrupted with rotational-acceleration mTBI.^1^ It has been proposed that this process might be part of a cascade that increases the risk for neurodegenerative disorders.^2,3^ In all severities of TBI, axonal tracts can stretch/tear to result in diffuse axonal injury and cause cellular functions to become dysregulated.^4^ During acute and subacute stages of TBI (mTBI and more severe injuries), protein markers for neuronal, axonal, and astrocyte damage as well as immune reactions have all been found to be increased compared to non-injured individuals.^5–8^ Many of the protein markers that increase after TBI have also been shown to increase in neurodegenerative disorders.^9–11^ While such markers appear to normalize within months after a TBI, there is some evidence that TBI might relate to greater pathological changes within the brain such as increased deposition of amyloid-β (Aβ) aggregates, neocortical Lewy bodies, and microinfarcts.^12,13^ Although not all studies have found a link between TBI and pathological outcomes, multiple lines point to a history of TBI being associated with an accelerated onset of cognitive impairment later-in-life in some individuals.^14–24^ Despite mixed findings in the literature, which might be explained by differences across studies in the definition of TBI and/or potential moderating factors present prior to injury (i.e., apolipoprotein E ɛ4 genetic susceptibility), an earlier onset of cognitive impairment has been independently linked to a history of TBI regardless of apolipoprotein E ɛ4 status, sex, and race.^18,25–30^ Thus, a prominent theory is that the neurobiological cascade of TBI may cause some adults with a history of TBI to have a higher burden of neurodegenerative-related changes, making them vulnerable to developing cognitive impairment earlier than individuals without a history of TBI. However, the specific links between mTBI and diagnoses of neurodegenerative conditions have mixed and unclear findings across clinical investigations.^31,32^ Moreover, there have been limited studies on whether prior mTBI might predispose persons to have higher pathophysiological markers related to neurodegenerative disorders later-in-life.

Alzheimer's disease pathophysiology is defined by aggregation of Aβ and tau proteins and evidence of neurodegeneration, which can be detected by measuring soluble Aβ_42_ (especially the ratio of Aβ_42/40_), total tau, and phosphorylated tau 181 (Ptau_181_) proteins within cerebrospinal fluid (CSF).^33^ We previously investigated if cognition and CSF markers of Aβ_42_, Ptau_181_, and total tau differed in a small sample having dementia of the Alzheimer's type (DAT) with and without a history of mTBI in the Alzheimer's Disease Neuroimaging Initiative dataset.^34^ Most had a single mTBI and were on average 30 years out from injury, and Ptau181 and total tau were observed to be nearly 33% higher for the mTBI group relative to the well-matched control group, with no meaningful differences on Aβ_42_ or cognition measures. Highly sensitive blood-derived assays have emerged with diagnostic and prognostic potential in neurodegenerative disorders as multiple markers have been associated with cognitive decline and postmortem neurodegenerative changes.^35^ However, when Aβ_40_, Aβ_42_, and tau along with neurofilament-light (NFL; marker of axonal injury) and glial fibrillary acidic protein (GFAP; marker of astrocytic response) were measured in plasma of cognitively intact former athletes having a history of mTBI, no differences in marker concentrations were observed in comparison to individuals with no history of mTBI.^36^ While the CSF-based findings may have been coincidence and mTBI might not influence pathophysiological markers, it is also possible that blood-derived content may be unsuitable for detecting mTBI effects in aged individuals or that links may only be noticeable at the mild cognitive impairment (MCI) and dementia stages. In either case, potential implications of mTBI during aging and risk for neurodegenerative conditions remain unclear, and possible mechanistic pathways for how mTBI could serve as a risk factor have been understudied. The present study was designed to explore the cross-sectional relationship between a history of mTBI and several blood-derived protein markers across the cognitive spectrum of normal cognition (NC), MCI, and DAT. We hypothesized that levels of blood-based biomarkers would be higher for individuals having a history of mTBI compared to those with no history of mTBI only at the stages of MCI and DAT.

## Methods

The Texas Alzheimer's Research and Care Consortium (TARCC; https://www.txalzresearch.org) dataset and biobank was utilized for this study.^37^ In brief, TARCC collected detailed clinical information, neuropsychological data (see Supplemental Material), and blood from research participants classified as NC, MCI, and DAT in similar fashion to NIA-funded Alzheimer Disease Research Centers. Blood was collected in EDTA tubes after an overnight fast, gently mixed, and centrifuged for 10 min at 1500 g to derive plasma specimens, from which 1 mL were aliquoted into polypropylene tubes and stored in −80°C freezers within 2 h of collection. Comprehensive information about neurotrauma exposure was obtained in a small cohort of TARCC participants using an interview similar to *The Ohio State University TBI Interview* method at the same visit in which blood and neuropsychological data were obtained.^38^ Participants were asked if they ever experienced a head injury which caused symptoms such as: loss of consciousness (LOC), feeling dazed/confused, headache, dizziness, balance problems, vision changes, mood alteration, or cognitive difficulties. Classification of prior mTBI was based on VA/DOD criteria and recorded as present when there was at least one injury resulting in brief LOC or an alteration in mental state during a participant's lifetime.^39^ Case-control matching was performed to derive mTBI + and mTBI- groups for this study (Supplemental Material).

Plasma samples underwent zero freeze-thaw cycles prior to biomarker measurement. Specimens were thawed, centrifuged for 5 min at 10,000 g to remove any debris, and diluted following the assay kit guidelines (4-fold dilution). Aβ_40_, Aβ_42_, Ptau_181_, tau (N-terminal to mid-domain), Transactive response DNA binding protein of 43 kDa (TDP43), NFL, and GFAP were measured on a single-molecule enzyme-linked immunoarray (SIMOA) HD-1 Analyzer (Quanterix, Lexington, MA). Protein concentrations were analyzed in duplicate following the manufacturer's instructions for the Neurology 4-Plex E, Tau 2.0, Ptau_181_ Advantage V2.1, and TDP-43 assays.

### Statistical analysis

Frequency distributions were inspected, and four of the six markers were skewed (Ptau_181_, Tau, NFL, GFAP) and log_2_ transformed for analyses. Analysis of variance using robust standard errors (HC3) to account for the matched nature of the sample compared biomarker concentrations between mTBI + and mTBI- individuals separately for each cognitive group (NC, MCI, DAT). Given sample sizes were limited for observing statistical significance in this exploratory study, effect sizes for comparisons were determined using Cohen's *d*. Statistical significance was corrected for multiple comparisons based on the Benjamini Hochberg false discovery rate (FDR) correction and the adjusted alpha level was set at 0.0028.

## Results

A total of 16 participants with NC (mTBI + n = 9; mTBI- n = 7), 12 participants with MCI (mTBI + n = 8; mTBI- n = 4), and 17 participants with DAT (mTBI + n = 6; mTBI- n = 11) were identified through case-control matching and selected for this exploratory study. Characteristics of the groups across the cognitive spectrum are displayed in Table 1. Among all mTBI + individuals, the majority reported only a single injury (72.7%) and no exposure to repeated head impacts (91.1%), and 56.5% had an injury causing loss of consciousness (all <10 min). The average time since injury(s) was 43.10 years (range = 12–69 years).

**Table 1.** Characteristics of participants across the cognitive spectrum.

	Normal Cognition			MCI			DAT		
Characteristics	mTBI- (n = 7)	mTBI+ (n = 9)	*p*	mTBI- (n = 4)	mTBI + (n = 8)	*p*	mTBI- (n = 11)	mTBI + (n = 6)	*p*
Age, M (SD)	68.6 (6.4)	66.7 (7.4)	0.30	74.8 (5.3)	72.3 (8.1)	0.30	78.0 (9.1)	81.5 (3.4)	0.14
Education, y, M (SD)	16.9 (1.9)	15.4 (2.4)	0.22	15.0 (2.6)	16.9 (2.5)	0.13	15.5 (2.5)	16.5 (2.7)	0.22
Female, No.	2	3	1.00	2	0	0.09	4	1	0.60
Non-White Race, No.	1	3	0.59	0	1	1.00	1	0	1.00
Hispanic, No.	0	1	1.00	0	1	1.00	0	0	-
HTN, No.	2	4	0.32	2	3	1.00	6	2	1.00
HCL, No.	3	3	1.00	3	4	0.49	6	3	0.60
Diabetes, No.	0	1	1.00	1	0	0.33	2	0	0.52
A-Fib, No.	0	1	1.00	1	3	0.55	0	0	−
Prior MI, No.	0	0	−	1	0	0.33	3	1	1.00
Prior Stroke, No.	0	0	−	0	0	−	0	0	−
*APOE4* Carrier, No.	3	4	1.00	0	2	0.44	6	3	1.00
Plasma Conc, M (SD)									
Aβ_42/40_	0.058 (0.008)	0.058 (0.007)	0.97	0.0556 (0.005)	0.057 (0.010)	0.81	0.054 (0.013)	0.048 (0.008)	0.24
Ptau_181_	29.03 (8.32)	32.00 (14.63)	0.78	31.52 (9.68)	43.33 (23.05)	0.32	45.79 (16.92)	53.30 (16.06)	0.33
Tau	1.06 (0.30)	1.41 (1.25)	0.72	1.08 (0.44)	1.06 (0.41)	0.98	1.07 (0.62)	1.04 (0.15)	0.44
TDP43	2276.51 (1098.20)	2783.68 (1345.65)	0.45	3280.14 (1355.71)	3520.21 (1634.46)	0.82	1922.17 (1286.99)	2392.10 (1425.58)	0.54
NFL	16.27 (5.94)	19.69 (15.42)	0.93	26.43 (27.66)	21.39 (12.77)	0.93	28.53 (11.90)	36.44 (8.60)	0.11
GFAP	78.04 (24.63)	80.37 (33.70)	0.98	99.18 (61.02)	98.55 (39.55)	0.95	172.89 (73.64)	189.93 (58.79)	0.50

Plasma values are mean raw concentrations measured in pg/mL. Raw values for Aβ_42/40_ and TDP43 were used in analyses, whereas the Log2 transformation of Ptau_181_, Tau, NFL, and GFAP were used instead given skewed distributions. A-Fib: atrial fibrillation; HTN: hypertension; HCL: hypercholesterolemia; MI: myocardial infarction.

Age, education, race/ethnicity, medical comorbidities, and *APOE* ε4 status were similar between mTBI + and mTBI- groups. Neuropsychological performances are displayed in Supplemental Table 1 and were similar between mTBI + and mTBI- individuals for the NC and MCI groups. In contrast, mTBI + DAT individuals had slightly poorer scores than mTBI- DAT individuals on measures of global cognition, episodic memory, and language, though no differences were statistically significant (q > 0.002).

**Figure 1.**
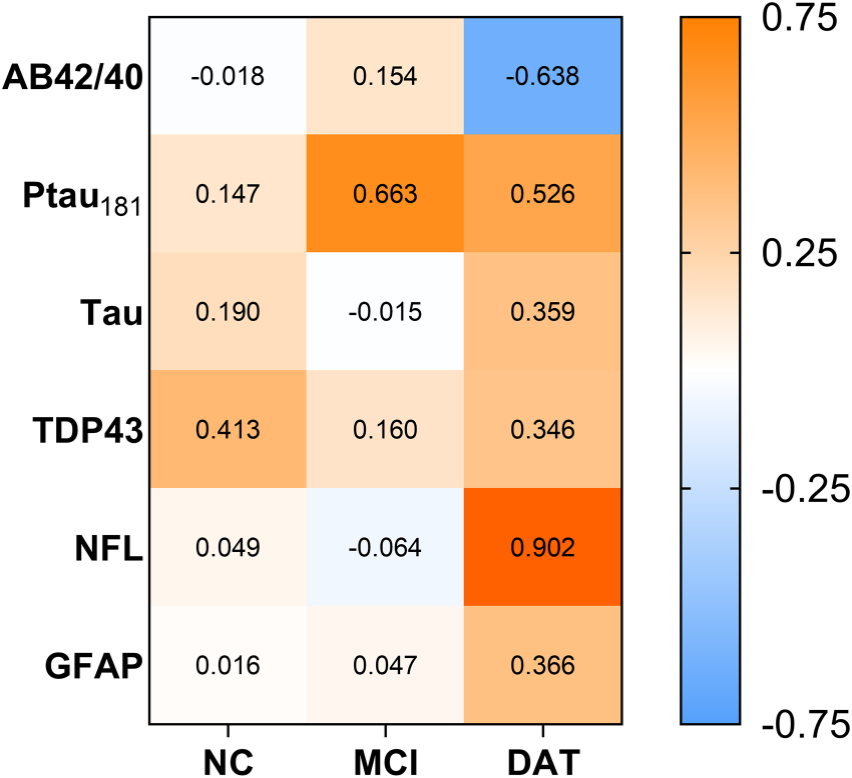
Effect size difference in plasma markers between mTBI + and mTBI- groups. Values are Cohen's d calculations. Positive values depict increased levels for mTBI + groups. Negative values depict decreased levels for mTBI + groups. NC: normal cognition; MCI: mild cognitive impairment; DAT: dementia of the Alzheimer's type.

Plasma protein levels were not statistically different between groups (*ps* 0.11–0.98). However, examination of effect sizes showed a medium effect size difference for higher Ptau_181_ in mTBI + individuals with MCI and DAT and medium-to-large effect sizes for lower Aβ_42/40_ and higher NFL in mTBI + persons with DAT, whereas there were no medium/large effect size differences in NC (Figure 1). mTBI + individuals had in comparison to mTBI- individuals: 16–33% higher Ptau_181_ in MCI and DAT, 28% higher NFL in DAT, and 11% lower Aβ_42/40_ in DAT.

## Discussion

This exploratory study is the first of its kind to examine the influence of a history of mTBI on neurodegenerative plasma markers across the cognitive continuum of NC, MCI, and DAT. Although comprised of small samples, the groups were well-matched and similar on many factors that could influence biomarker levels (e.g., age, medical comorbidities), making the findings informative and important.^40^ Statistical significance testing did not demonstrate differences between mTBI + and mTBI- groups for any markers. This is not surprising given statistical results (i.e., p-values) are greatly influenced by sample size, and why the use of effect size measures have been emphasized for scientific investigation.^41–43^ Along these lines, our exploratory study found medium-to-large effect sizes for mTBI + groups having higher Ptau_181_ in MCI and DAT alongside higher NFL and lower Aβ_42/40_ in DAT. Thus, plasma-derived markers may have promise for detecting associations of a history of mTBI with the biological processes involved in Alzheimer's disease and related disorders. Increased plasma Ptau_181_ parallels the increased levels in CSF seen in DAT in our prior study, where individuals were well-matched on disease stage and many other factors, reinforcing the possibility that a mTBI decades ago might be associated with increased pathophysiological burden to serve as a risk factor for neurodegenerative disorders.^34^

It is unknown whether TBI and elevated Ptau_181_ or other markers are connected on a pathophysiological level. Protein markers have a short half-life in biofluids, and thus, any abnormalities suggest “active” pathophysiological processes that either resolve or persist over time.^44,45^ While Ptau_181_ and NFL have been shown to increase after mTBI, levels normalized within days/months.^6,46^ As such, it could be hypothesized that biomarkers might increase as a delayed consequence of injury. This may arise from an injury provoking a chronic alteration of cellular functions (e.g., inflammation, oxidative stress, etc.) potentially leading to downstream pathophysiological changes seen in neurodegenerative disorders. Alternatively, ongoing research in the field is investigating the possibility that immune memory mechanisms involving the innate and adaptive immune system could play a role in excessive responses to future brain insults, such as those seen in neurodegenerative disorders, potentially inducing toxic effects.^47^

With no biomarker differences evident in NC, the impact of a history of mTBI on soluble biomarkers currently available might be noticeable only at the MCI and dementia stages. New markers that could detect biological abnormalities upstream of changes in Aβ_42/40_, Ptau_181_, and the others examined, or measure only brain-specific processes, may be necessary at the NC stage to see possible vulnerabilities linked to prior mTBI. Alternatively, there could be differences in the trajectory, timing, or order of biomarker changes related to a history of mTBI that might be more easily identified among those with NC. Thus, future studies are needed replicating the current results in large samples and exploring if a history of mTBI influences the temporal evolution of markers in NC on route to manifesting cognitive decline.

This study has several strengths, including having detailed information on head impact exposure for group assignment and use of ultrasensitive commercially available blood assays. However, the study has limitations beyond small sample sizes that warrant consideration. A reliance on self or informant-report methods for mTBI, as with many aging/dementia registries, allows potential for recall bias. Furthermore, biomarker information to characterize positivity/negativity of Alzheimer's pathology in participants was not available. We also cannot exclude the possibility that chronic kidney disease, a medical condition unavailable in the dataset shown to influence blood-based marker levels, may have been present in some participants and relate to the higher marker concentrations observed.^48,49^ As a result, this exploratory study extends earlier findings that neurodegenerative markers may be increased by a history of mTBI and provides preliminary evidence that such associations are detectable in plasma, but additional research in larger samples will be necessary.

## Supplemental Material

sj-docx-1-alz-10.1177_13872877251325757 - Supplemental material for A preliminary study on plasma markers across cognitive stages and links to a history of mild traumatic brain injury Supplemental material, sj-docx-1-alz-10.1177_13872877251325757 for A preliminary study on plasma markers across cognitive stages and links to a history of mild traumatic brain injury by Christian LoBue, Barbara E Stopschinski, Nil Saez Calveras, Amber Salter, Doug Galasko, Chris Giza, C Munro Cullum and Peter M Douglas, John Hart in Journal of Alzheimer's Disease
