# Supplementary material for: A preliminary study on plasma markers across cognitive stages and links to a history of mild traumatic brain injury
Source: J Alzheimers Dis. 2025 Mar 21;105(1):49–55. doi: 10.1177/13872877251325757 (PMC12055478; doi:10.1177/13872877251325757)
Supplement: sj-docx-1-alz-10.1177_13872877251325757 - Supplemental material for A preliminary study on plasma markers across cognitive stages and links to a history of mild traumatic brain injury [file sj-docx-1-alz-10.1177_13872877251325757.docx]

**Supplemental Material**

**A preliminary study on plasma markers across cognitive stages and links to a history of mild traumatic brain injury**

**Cognitive classification**

Participants were classified as having NC, MCI, or DAT through a consensus conference using established clinical criteria and reviewing data from a clinical interview, neurological exam, and neuropsychological testing.^1,2^

**Case-control matching and participant selection**

Case-control matching without replacement was performed from a larger sample of 100 individuals to derive mTBI+ and mTBI- groups. Exact matching was completed for clinical classification, and mTBI- participants were additionally selected through age and years of education matching with a tolerance of ± 4 years to mTBI+ participants.

**Neuropsychological assessment**

Each participant completed a brief neuropsychological battery consisting of the CERAD Word Learning Memory Test, Boston Naming Test-Short Form, verbal fluency, Southwestern Assessment of Processing Speed, and Trail Making Test Part B.^3–8^

**Mild TBI history interview**

Participants were asked if they ever experienced a head injury which caused symptoms such as: LOC, feeling dazed/confused, headache, dizziness, balance problems, vision changes, mood alteration, or cognitive difficulties. Seven related questions were asked when prior neurotrauma was endorsed: 1) number of injuries with LOC, 2) duration of each injury with LOC, 3) number of injuries without LOC, 4) age of injury(s), 5) exposure to multiple head impacts without symptoms, 6) situation of injury(s) and repeated head impacts, and 7) duration to recovery for worst injury.

***APOE4* genotyping**

Apolipoprotein E genotyping was completed as previously described.^9^

**Biomarker measurement**

Pooled intra-assay coefficients of variation were 3.83% for Aβ_40_, 4.14% for Aβ_42_, 9.20% for Ptau181, 8.77% for tau, 11.04% for TDP43, 5.59% for NFL, and 5.43% for GFAP.

**References**

1. Albert MS, DeKosky ST, Dickson D, et al. The diagnosis of mild cognitive impairment due to Alzheimer’s disease: recommendations from the National Institute on Aging‐Alzheimer’s Association workgroups on diagnostic guidelines for Alzheimer’s disease. *Alzheimers Dement* 2011; 7: 270–279.

2. McKhann GM, Knopman DS, Chertkow H, et al. The diagnosis of dementia due to Alzheimer’s disease: recommendations from the National Institute on Aging‐Alzheimer’s Association workgroups on diagnostic guidelines for Alzheimer’s disease. *Alzheimers Dement* 2011; 7: 263–269.

3. Moms JC, Heyman A, Mohs RC, et al. The Consortium to Establish a Registry for Alzheimer’s Disease (CERAD). Part I. Clinical and neuropsychological assessment of Alzheimer’s disease. *Neurology* 1989; 39: 1159–1159.

4. Williams BW, Mack W, Henderson VW. Boston Naming Test in Alzheimer’s disease. *Neuropsychologia* 1989; 27: 1073–1079.

5. Spreen O and Benton A. *Neurosensory Center Comprehensive Examination for Aphasia*. Victoria, BC: Neuropsychological Laboratory, University of Victoria, 1977.

6. Goodglass H and Kaplan E. *Boston Diagnostic Aphasia Examination (BDAE)*. Philadelphia, PA: Lea and Febiger, Psychological Assessment Resources, 1983.

7. Cullum CM, Galusha JM, Wadsworth HE, et al. Southwestern Assessment of Processing Speed (SWAPS): A new brief test with demographically-corrected norms in an ethnically and educationally diverse population. *Clin Neuropsychol* 2022; 36: 2260–2277.

8. Partington JE and Leiter RG. Partington’s Pathways Test. *Psych Serv Center Bull* 1949; 1: 11–20.

9. Waring SC, O’Bryant SE, Reisch JS, et al. The Texas Alzheimer’s Research Consortium longitudinal research cohort: Study design and baseline characteristics. *Tex Public Health J* 2008; 60: 9–13.

**Supplemental Table 1.** Neuropsychological scores between groups.

| \|  \| \| \| \| \| \| \|  \| \|  \| \| \| --- \| --- \| --- \| --- \| --- \| --- \| --- \| --- \| --- \| --- \| --- \| \| **Groups** \| **MMSE**  Median IQR \| **CERAD TL**  Median IQR \| **CERAD DR**  Median IQR \| **BNT**  Median IQR \| **Letter Fluency**  Median IQR \| **Semantic Fluency**  Median IQR \| \| **SWAPS**  Median IQR \| \| \| NC mTBI- \| 29.0 27-30 \| 22.0 19-25 \| 9.0 7-10 \| 29.0 28-30 \| 49.0 38-50 \| 23.0 18-33 \| \| 33.0 30-39 \| \| \| NC mTBI+ \| 29.0 28-30 \| 19.0 18-25 \| 7.0 6-0 \| 28.0 27-30 \| 45.0 27-52.5 \| 17.0 14.5-22.5 \| \| 34.0 29.3-40.8 \| \| \|  \|  \|  \|  \|  \|  \|  \| \|  \| \| \| MCI mTBI- \| 28.5 25.8-29 \| 15.0 11.8-19 \| 6.0 3.3-8 \| 26.5 26-29.3 \| 26.0 18.5-39.5 \| 14.0 10.3-20.8 \| \| 24.5 20.5-29.3 \| \| \| MCI mTBI+ \| 27.0 26.3-28.8 \| 14.5 12.3-16.5 \| 5.0 4-6.8 \| 29.0 23.5-29 \| 29.0 25.8-39.8 \| 18.0 14.8-21 \| \| 25.0 17.5-29.5 \| \| \|  \|  \|  \|  \|  \|  \|  \| \|  \| \| \| AD mTBI- \| 22.0 17-23 \| 10.0 8.5-14 \| 1.5 0-3 \| 22.0 17-26 \| 28.0 18-48 \| 12.0 10-12 \| \| 18.0 14.8-20 \| \| \| AD mTBI+ \| 19.5 15-23.3 \| 7.5 2.8-9.5 \| 0.0 0-1.3 \| 19.0 14-25.8 \| 31.5 16-37.8 \| 9.5 7.5-15 \| \| 21.0 16-23 \| \| |
| --- | --- | --- | --- | --- | --- | --- | --- | --- | --- | --- | --- | --- | --- | --- | --- | --- | --- | --- | --- | --- | --- | --- | --- | --- | --- | --- | --- | --- | --- | --- | --- | --- | --- | --- | --- | --- | --- | --- | --- | --- | --- | --- | --- | --- | --- | --- | --- | --- | --- | --- | --- | --- | --- | --- | --- | --- | --- | --- | --- | --- | --- | --- | --- | --- | --- | --- | --- | --- | --- | --- | --- | --- | --- | --- | --- | --- | --- | --- | --- | --- | --- | --- | --- | --- | --- | --- | --- | --- | --- | --- | --- | --- | --- | --- | --- | --- | --- | --- | --- | --- | --- |

No statistical differences using False Discovery Rate correction. MMSE: Mini-Mental State Examination; CERAD TL: CERAD Word Learning Memory Test Total Learning; CERAD DR: CERAD Word Learning Memory Test Delayed Recall; BNT: Boston Naming Test; SWAPS: Southwestern Assessment of Processing Speed
